# Determination of InN/Diamond Heterojunction Band Offset by X-ray Photoelectron Spectroscopy

**DOI:** 10.1007/s11671-010-9796-6

**Published:** 2010-09-30

**Authors:** K Shi, DB Li, HP Song, Y Guo, J Wang, XQ Xu, JM Liu, AL Yang, HY Wei, B Zhang, SY Yang, XL Liu, QS Zhu, ZG Wang

**Affiliations:** 1 Key Laboratory of Semiconductor Materials Science, Institute of Semiconductors, Chinese Academy of Sciences, P. O. Box 912, 100083, Beijing, People's Republic of China; 2 Key Laboratory of Excited State Processes, Changchun Institute of Optics, Fine Mechanics and Physics, Chinese Academy of Sciences, 16 Dong Nan Hu Road, 130033, Changchun, People's Republic of China

**Keywords:** Valence band offset, InN/diamond heterojunction, X-ray photoelectron spectroscopy, Conduction band offset

## Abstract

Diamond is not only a free standing highly transparent window but also a promising carrier confinement layer for InN based devices, yet little is known of the band offsets in InN/diamond system. X-ray photoelectron spectroscopy was used to measure the energy discontinuity in the valence band offset (VBO) of InN/diamond heterostructure. The value of VBO was determined to be 0.39 ± 0.08 eV and a type-I heterojunction with a conduction band offset (CBO) of 4.42 ± 0.08 eV was obtained. The accurate determination of VBO and CBO is important for the application of III-N alloys based electronic devices.

## Introduction

Among the group III nitrides, InN is of great interest because of its extremely high predicted electron mobility [1], small effective mass [2,3], and large electron saturation drift velocity [4]. With the latest progress in improving the film quality these years, InN film has been considered to be able to meet the requirements for application to practical devices [5,6]. It is expected to be a highly promising material for the fabrication of high performance,high electron mobility transistor (HEMT) due to its electronic properties. Moreover, the re-evaluation of the InN bandgap and subsequent findings [7,8] have opened up interesting opportunities for using InN in new applications, such as high-efficiency solar cells [9], solid state lighting [10-12], and 1.55 μm emission for fiber optics [13,14]. As the hardest material with high optical transparency from ultraviolet to infrared range, diamond is an excellent transparent window for InN based photoelectric devices mentioned above. It can also be used as lens coatings for infrared transmissions. The bandgap of diamond at room temperature is ~5.45 eV, so it is a promising carrier confinement layer for InN based HEMT, which requires a larger bandgap barrier to confine electrons. Furthermore, because of the combination of its unique electronic and thermal properties, diamond plays a vital or somewhat irreplaceable role in some special applications, such as in abominable environments and military fields. Up to now, the GaN/diamond system has already been studied by a lot of groups [15,16]. However, there is lack of experimental data available on the interface band alignment parameters for InN/diamond system. X-ray photoelectron spectroscopy (XPS) has been demonstrated to be a direct and powerful tool for measuring the valence band offsets (VBOs) of heterojunctions [6,17-19]. In this letter, we report an experimental measurement of the VBO in InN/diamond heterojunction by XPS.

## Experimental

Three samples were used in our XPS experiments, namely, a 350-nm-thick InN layer grown on c-plane sapphire, a 2-mm-thick single-crystal diamond synthesized at high temperature and high pressure(HTHP), and a ~5-nm-thick InN grown on diamond. InN films in this study were grown by horizontal low-pressure metal-organic chemical vapor deposition, as reported elsewhere [19].The crystal structures were characterized using the high-resolution X-ray diffraction (HRXRD) apparatus at Beijing Synchrotron Radiation Facility (BSRF). The incident X-ray beam is monochromized to 0.154791 nm by a Si (111) monocrystal. According to the XRD results, single-crystal diamond (400) and wurtzite InN (002) were obtained. Both diamond and InN in our experiment are undoped, while the InN films are unintentionally n-type doped, with carrier concentration and Hall mobility being 6.2 × 10^19^ cm^-3^ and 370 cm^2^/Vs respectively, as determined by Hall effect measurement in the InN/sapphire film.

The XPS measurements were performed on a PHI Quantera SXM instrument with Al Kα (energy 1486.6 eV) as the X-ray radiation source, which had been carefully calibrated utilizing work function and Fermi energy level (*E*_F_). A large number of electrons are excited and emitted from the sample during the test, so the sample is always positively charged and the consequent electric field can affect the measured kinetic energy of photoelectron. A low-energy electron flood gun was utilized to achieve charge compensation. The total energy resolution of this XPS system is about 0.5 eV, and the accuracy of the observed binding energy is within 0.03 eV after careful calibration. The measurements were as follows: first, low-resolution survey scan mode was used to determine which elements were present on the sample surfaces. Then, very-high-resolution spectra were acquired to determine the binding energy (i.e., chemical state) in the survey spectra. Since only the relative energy position in each sample is needed to determine the VBO, the absolute energy calibration for a sample has no effect on the universal energy reference.

## Results and Discussion

The VBO (Δ*E*_*v*_) can be calculated from the formula

**Figure 1:**
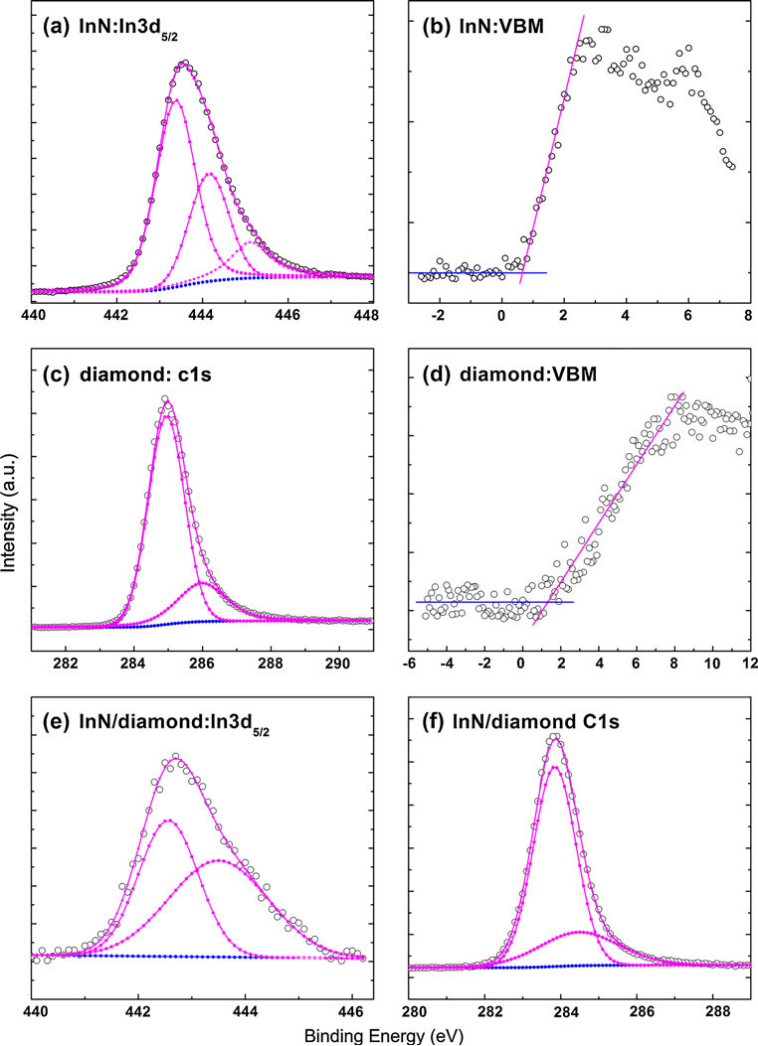
**In 3d_5/2_ Core level XPS spectra for a InN and e InN/diamond samples, and C1*s* XPS spectra for c diamond and f InN/diamond samples**. **b** InN and **d** diamond are the valence band spectra. All peaks have been fitted using a Shirley background and Voigt (mixed Lorentzian-Gaussian) line shapes, as summarized in Table 1.

(1) ΔEV=ΔECL−(EIn3d5/2InN−EVBMInN)+(EC1sdiamond−EVBMdiamond)

where $\Delta E_{\text{CL}}=\left(E_{\text{In}3{\text{d}}_{5/2}}^{\text{InN}}-E_{\text{C}1s}^{\text{diamond}}\right)$ is the energy difference between In3d_5/2_ and C1*s* core levels (CLs) in InN and diamond, which are measured in the InN/diamond heterojunction. $\left(E_{\text{In}3{\text{d}}_{5/2}}^{\text{InN}}-E_{\text{VBM}}^{\text{InN}}\right)\;$ and $\;\left(E_{\text{C}1s}^{\text{diamond}}-E_{\text{VBM}}^{\text{diamond}}\right)$ are the InN and diamond bulk constants respectively, measured from the two corresponding thick films. VBM stands for valance band maximum. The In3d_5/2_ spectra for the InN and InN/diamond samples, the C1*s* spectra for the diamond and InN/diamond samples, and the valence band photoemission for both InN and diamond samples are shown in Figure 1. All peaks have been fitted using a Shirley background and Voigt (mixed Lorentzian-Gaussian) line shapes. The position of the VBM with respect to the surface Fermi level was determined by the intersection of linear fitting to the leading edge of the valence band photoemission and the background [20]. All the parameters deduced from Figure 1 are summarized in Table 1 for clarity.

**Table 1:** XPS CL spectra fitting results and VBM positions obtained by linear extrapolation of the leading edge to the extended base line of the VB spectra

Sample	State	Binding energy (eV)	Bonding	FWHM (eV)
Diamond	C1 *s*	284.90	C–C	1.21
		286.00	C–O	1.89
	VBM	1.32	–	
InN	In 3d_5/2_	443.42	In–N (screened)	1.09
		444.21	Adsorbed In–O	1.09
		445.27	In–N (unscreened)	2.45
	VBM	0.66	–	
InN/diamond	In 3d_5/2_	442.59	In–N (screened)	1.26
		443.50	In–N (unscreened)	2.19
	C1 *s*	283.80	C–C (screened)	1.28
		284.50	C–C (unscreened)	2.43

All the binding energies are referenced to the Fermi level (0 eV)

As illustrated in Figure 1a, e, the In3d_5/2_ core-level lineshapes are slightly asymmetric, with a high binding energy shoulder on the main peaks. This phenomenon has been reported by several groups [20-22]. Due to the high carrier density in unintentionally n-type doped InN and the surface electron accumulation effect, the photoemitted electrons will lose energy by coupling with the free electron plasmas at the surface of the samples. As we know, plasmons lead to the quantization of a collective excitation of the electron gas in a solid. In metals, however, Plasmon satellites are commonly observed in photoemission spectra of core-level peaks on the high binding energy side. The unrelexed Koopaman's state produced by removal of a core electron is not an eigenstate and is projected onto "screened" and "unscreened" final eigenstates [21], the latter corresponding to a plasmon satellite at higher binding energies than the screened state. The unscreened final state usually gives a peak with a broader Lorentzian peak profile whose width reflects the plasmon lifetime, which in turn depends on the conduction electron relaxation time [21]. According to this, we attribute the component with lower binding energy and smaller half-width to "screened" final-state, while that with higher binding energy and broader half-width to "unscreened" final-state, as is shown in Table 1. Indeed, the "unscreened" higher-binding energy components are much broader than the "screened" lower-binding energy components in our XPS spectra. Similar plasmon loss features have also been observed in the materials SnO_2_ when heavily doped with Sb [23,24], indium-tin-oxide [21] and PbO_2 _[25]. Wertheim [26,27] calculated the influence of surface plasmon to binding energy in narrow band metal, from which we can estimate that influence to our system. In Wertheim's model, the surface plasmon energy, designated as $\hbar \omega_{\text{sp}}$ is considered to be

(2) $$\hbar \omega_{\text{sp}}={\left(\frac{ne^{2}}{\left(\varepsilon \left(\infty \right)+1\right)\varepsilon_{0}m^{*}}\right)}^{1/2}$$

Here *n* is the carrier concentration, ε(∞) is the high frequency dielectric constant, and m* is the effective mass of the conduction electrons. Compared with the metals in his model, the carrier concentration in our sample surface is much lower, which means that only small energy separations exist between the screened and unscreened core-level components. This results in an asymmetric core-level XPS lineshape with just a weak high binding energy tail due to plasmon losses, which is consistent with our experimental results.

Based on all the arguments made above, we attribute the lower-binding energy component (443.42 eV) in Figure 1a to the "screened" final-state peak in In3d_5/2_ photoemission, the higher-binding energy component (445.27 eV) to the "unscreened" final-state peak, and the mid-binding energy component (444.21) to the In–O bonding. In Figure 1c, the lower-binding energy component (284.9 eV) and the higher-binding energy component (286.00 eV) are considered to be C–C bonding and C–O bonding respectively [28,29].

In order to avoid the surface oxidation and reduce the contamination effect, the InN/diamond sample was subjected to a surface clean procedure by Ar^+^ bombardment with a voltage of 1 kV at a low sputtering rate of 0.5 nm/min, which alleviates damages to the sample. The reduced thickness (less than 1 nm) is calculated by the sputtering rate, and the O-related bondings were absent in cleaned InN/diamond heterojunction because of the sputtering process. In Figure 1e, the lower-binding energy component (442.59 eV) and the higher-binding energy component (443.50 eV) are attributed to be screened In–N bonding and unscreened In-N bonding respectively. Finally, in Figure 1f, we suggest assignments of screened C–C bonding and unscreened C–C bonding for the lower- (283.80 eV) and higher-binding (284.50 eV) energy components, respectively. The VBM of the two thick samples are determined to be 0.66 and 1.32 eV, respectively. All of them are summarized in Table 1.

**Figure 2:**
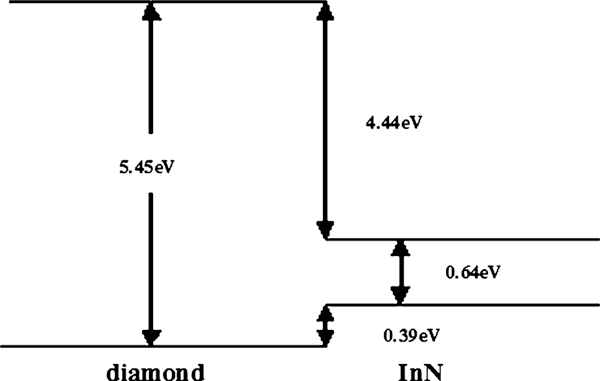
**The VBM and CBM line-up of InN/diamond heterojunction at room temperature**. A type-I band heterojunction is formed in straddling configuration.

The lower-binding energy components related to "screened" final-state are chosen for VBO calculation because the peak and line width of higher-binding energy ("unscreened" final-state) depend on the excitation of bulk, surface plasmon, and surface treatment [22,23]_,_ as is mentioned above. The VBO values can be calculated by substituting those measured values in Table 1 into Eq. 1. The average InN/diamond VBO (Δ*E*_*v*_) is -0.39 ± 0.08 eV. The CBO (Δ*E*_*C*_) is given by the formula $\Delta E_{C}=\left(E_{g}^{\text{diamond}}-E_{g}^{\text{InN}}\right)-\Delta E_{V}$. Here $E_{g}^{\text{diamond}}$ (~5.45 eV) and $E_{g}^{\text{InN}}$ (~0.64 eV) are respectively the bandgap of diamond and InN at room temperature. So the band lineup can be determined, with a conduction band offset (CBO) of 4.42 ± 0.08 eV, as shown in Figure 2.

As XPS measurements are spatially averaged due to the finite mean free path of elastic electrons (1.5–2 nm), band bending could induce a systematic error in our measurements. Due to the lattice mismatch between InN and diamond, especially the small linear pressure coefficient of InN (~0.06 meV/GPa) [30], the band gap change induced by the interface strain could be neglected. So the systematic error related to band bending is expected to be much smaller than the average standard deviation of 0.08 eV given above. Another factor that may affect the precision of the VBO value is the strain-induced piezoelectric field in the overlayer of the heterojunction, as described in the III-nitrides system [31]. By using the constants and equation in Martin's work [31], the field magnitude is estimated to be in the order of 10^7^ V/m. Assuming the heterojunction InN overlayer thickness of ~4 nm after Ar^+^ bombardment, the error of VBO induced by lattice mismatch is less than 60 meV. Besides, practically all nitride epitaxial layers are characterized by dense networks of threading defects extending from the substrates to the surfaces [31]_,_ the strains in pseudomorphic epi-films relieve mostly, which means the "residual" effect of piezoelectric field is greatly reduced. As a result, the strain-induced piezoelectric effect can be neglected here.

## Conclusions

In summary, the valence band offset of the InN/diamond heterojunction has been measured by XPS. A type-I band alignment with a valence band offset of Δ*E*_*v*_ ~ 0.39 ± 0.08 eV and conduction band offset of Δ*E*_*c*_ ~ 4.42 eV was obtained. The accurate determination of the band alignment of InN/diamond indicates that the diamond can provide an effective carrier confinement in InN/diamond based electronic devices.
